# Hyperglycemia exacerbates acetaminophen-induced acute liver injury by promoting liver-resident macrophage proinflammatory response via AMPK/PI3K/AKT-mediated oxidative stress

**DOI:** 10.1038/s41420-019-0198-y

**Published:** 2019-07-19

**Authors:** Qi Wang, Song Wei, Haoming Zhou, Gefenqiang Shen, Xiaojie Gan, Shun Zhou, Jiannan Qiu, Chenyu Shi, Ling Lu

**Affiliations:** 1 Liver Transplantation Center, The First Affiliated Hospital of Nanjing Medical University, Key Laboratory of Liver Transplantation, Chinese Academy of Medical Sciences, NHC Key Laboratory of Living Donor Liver Transplantation, Nanjing, China; 2 School of Medicine, Southeast University, Nanjing, China

**Keywords:** Acute inflammation, Kupffer cells

## Abstract

Although diabetes mellitus/hyperglycemia is a risk factor for acute liver injury, the underlying mechanism remains largely unknown. Liver-resident macrophages (Kupffer cells, KCs) and oxidative stress play critical roles in the pathogenesis of toxin-induced liver injury. Here, we evaluated the role of oxidative stress in regulating KC polarization against acetaminophen (APAP)-mediated acute liver injury in a streptozotocin-induced hyperglycemic murine model. Compared to the controls, hyperglycemic mice exhibited a significant increase in liver injury and intrahepatic inflammation. KCs obtained from hyperglycemic mice secreted higher levels of the proinflammatory factors, such as TNF-α and IL-6, lower levels of the anti-inflammatory factor IL-10. Furthermore, enhanced oxidative stress was revealed by increased levels of reactive oxygen species (ROS) in KCs from hyperglycemic mice post APAP treatment. In addition, ROS inhibitor NAC resulted in a significant decrease of ROS production in hyperglycemic KCs from mice posttreated with APAP. We also analyzed the role of hyperglycemia in macrophage M1/M2 polarization. Interestingly, we found that hyperglycemia promoted M1 polarization, but inhibited M2 polarization of KCs obtained from APAP-exposed livers, as evidenced by increased MCP-1 and inducible NO synthase (iNOS) gene induction but decreased Arg-1 and CD206 gene induction accompanied by increased STAT1 activation and decreased STAT6 activation. NAC restored Arg-1, CD206 gene induction, and STAT6 activation. To explore the mechanism how hyperglycemia regulates KCs polarization against APAP-induced acute liver injury, we examined the AMPK/PI3K/AKT signaling pathway and found decreased AMPK activation and increased AKT activation in liver and KCs from hyperglycemic mice post APAP treatment. AMPK activation by its agonist AICAR or PI3K inhibition by its antagonist LY294002 inhibited ROS production in KCs from hyperglycemic mice post APAP treatment and significantly attenuated APAP-induced liver injury in the hyperglycemic mice, compared to the control mice. Our results demonstrated that hyperglycemia exacerbated APAP-induced acute liver injury by promoting liver-resident macrophage proinflammatory response via AMPK/PI3K/AKT-mediated oxidative stress.

## Introduction

Diabetes mellitus is a metabolic disorder characterized by hyperglycemia and high morbidity worldwide^1^. Studies revealed that hyperglycemia aggravated acute liver injury^2,3^. The detrimental effects of hyperglycemia include an increase in the oxidative stress response and an enhanced inflammatory response^1^. Acetaminophen (APAP) is a commonly used nonprescription analgesic and antipyretic in many developed countries^4^, accidental or intentional overdose with APAP results in lethal liver injury^5^. It is well established that APAP induces hepatic inflammation and liver cell damage^6^. Whether hyperglycemia aggravates APAP-induced acute liver injury remains largely unknown.

Kupffer cells (KCs) are reemerging as critical mediators of both liver injury and repair. KCs exhibit a tremendous plasticity, depending on the local metabolic and immune environment. They can express a range of polarized phenotypes, from the proinflammatory M1 phenotype to the anti-inflammatory M2 phenotype^7^. M1 macrophages are characterized by induction of STAT1 and NF-kB transcription factors, as well as production of proinflammatory cytokines including TNF-α, IL-1, IL-6, and IL-12^8^. M2 macrophages are characterized by activation of transcription factor STAT6, elevated expression of mannose receptor (CD206), and production of cytokines such as TGF-β, CCL18, and IL-1Ra^9^.

Oxidative stress is caused by intracellular presence of reactive oxygen species (ROS) that overcomes the natural anti-oxidant defense of the cell^10^. Studies had shown that oxidative stress was a major mediator that underlied diabetic complications^11,12^. Diabetic conditions trigger ROS formation in macrophages^13^. The diabetes-induced secretion of monocyte chemotactic protein CCL2 (MCP-1) by endothelial cells attracted monocytes, whereas upregulated endothelial surface expression of adhesion molecule VCAM promoted their adhesion and diapedesis^13^. Transmigrated monocytes differentiated into macrophages and further exacerbated inflammation by mediating tissue injury and secreting proinflammatory cytokines and proteases as well as producing increased ROS levels in the tissues^13^. Studies showed hyperglycemia were closely associated with the production of ROS^14,15^. Hyperglycemia could mediate retinopathy and kidney damage by inducing ROS production^14,15^. Hyperglycemia caused mitochondrial dysfunction in endothelial cells and macrophages and aberrant activation of cytoplasmic NADPH oxidases (NOX) that together exacerbated ROS production^16,17^. Whereas the mechanism that hyperglycemia increases the production of ROS in KCs against APAP-induced acute liver injury remains unclear.

In this study, we determined that hyperglycemia aggravated acute liver injury by promoting liver-resident macrophage proinflammatory response via AMPK/PI3K/AKT-induced oxidative stress.

## Results

### Hyperglycemia exacerbates APAP-induced acute liver injury

**Fig. 1:**
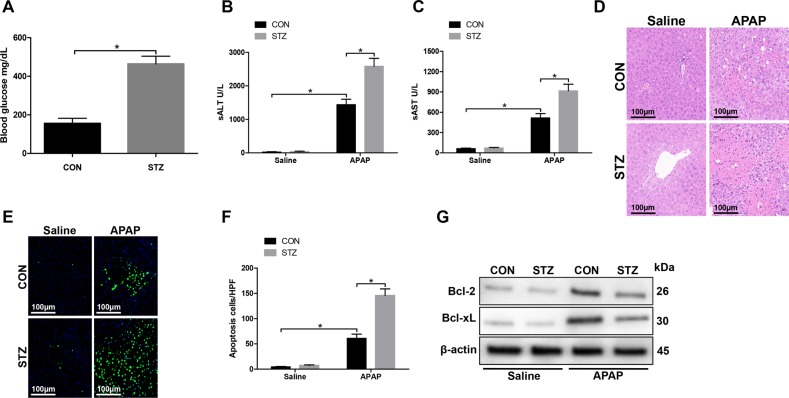
Hyperglycemia exacerbates APAP-induced acute liver injury. Hyperglycemic (streptozotocin, STZ) and control (CON) mice were prepared as described in “Materials and methods” section. APAP-induced acute liver injury or a sham procedure was performed. **a** Blood glucose levels were measured in both groups (*n* = 6/group). **b**, **c** Liver injury was evaluated 24 h post APAP treatment in terms of serum AST and ALT levels (*n* = 6/group). **d** Liver injury was evaluated in terms of liver histopathology. Representative of six mice/group. **e** TUNEL staining of liver sections (original magnification ×200). DAPI was used for nuclear staining. Representative of six mice/group. **f** The ratio of TUNEL-positive cells in different experimental groups (*n* = 6/group). **g** Bcl-2, Bcl-xL, and β-actin protein levels were measured by western blot. Representative of three experiments (mean ± SD, *p < 0.05)

To better study the role of diabetes/hyperglycemia in the development of APAP-induced acute liver injury, we induced type I diabetes in C57/B6J mice with multiple injections of low-dose STZ. Hyperglycemia was confirmed as shown in Fig. 1a. Indeed, compared with CON groups, mice in the STZ groups developed significantly more severe liver injury at 24 h post treated with APAP, as demonstrated by higher serum alanine aminotransferase (ALT) and aspartate transaminase (AST) levels (Fig. 1b, c), severely damaged liver architecture (Fig. 1d), extensive hepatocellular apoptosis (Fig. 1e, f), and significantly lower levels of antiapoptotic proteins Bcl-2 and Bcl-xL (Fig. 1g). Thus, our results indicated that hyperglycemia increased APAP-induced acute liver injury.

### Hyperglycemia enhances KC-related inflammation posttreated with APAP

**Fig. 2:**
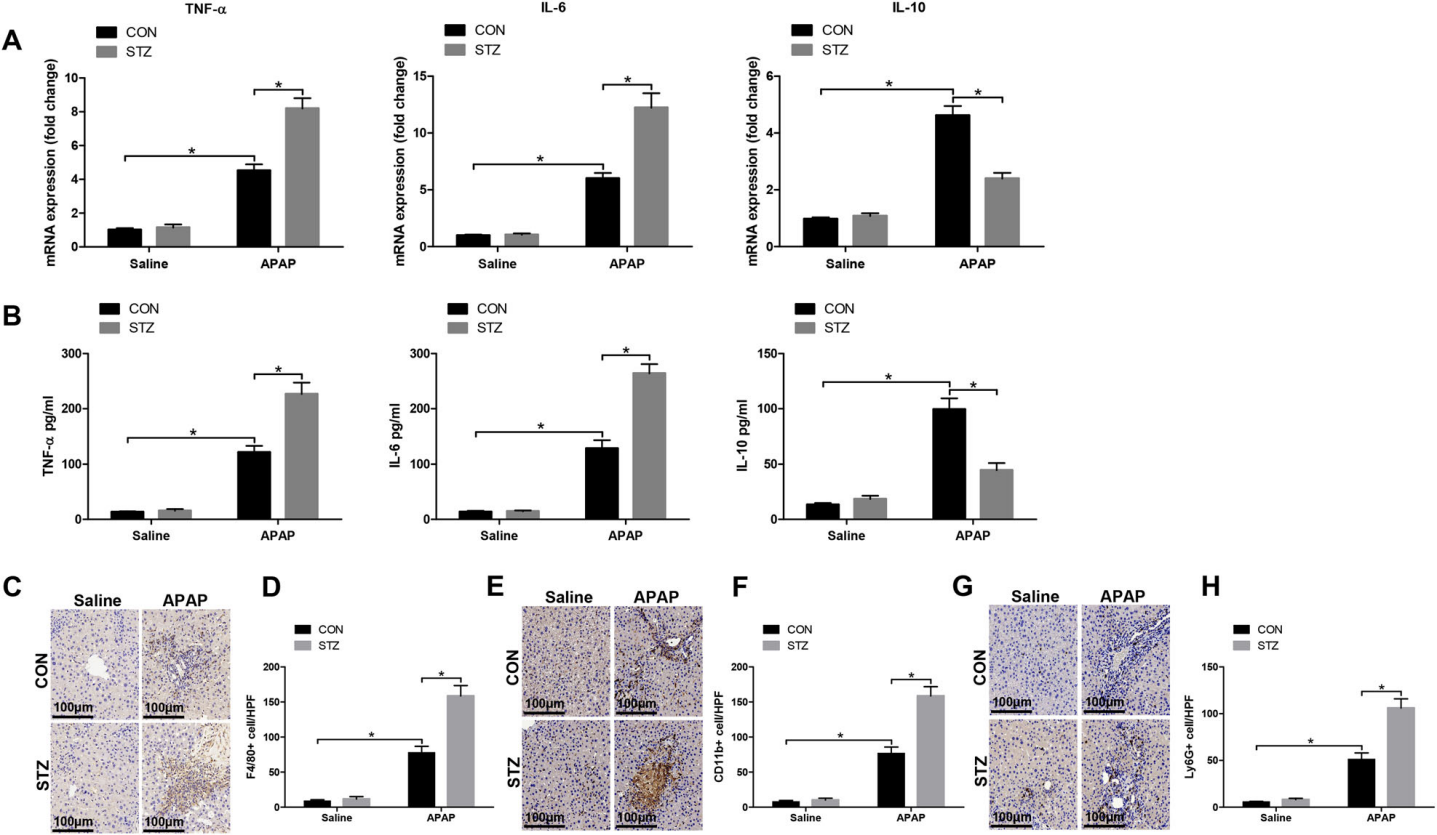
Hyperglycemia enhances KC-related inflammation response posttreated with APAP. Hyperglycemic (streptozotocin, STZ) and control (CON) mice were prepared as described in “Materials and methods” section. **a** APAP-induced acute liver injury or a sham procedure was performed. Inflammatory gene expression in liver was measured by quantitative RT-PCR (*n* = 6/group). **b** Serum levels of inflammatory cytokines were measured by ELISA (*n* = 6/group). **c-h** F4/80 + macrophages, CD11b + macrophages, and Ly6G + neutrophils infltration in liver was detected by immunohistochemical staining (original magnification ×200). Representative of six mice/group. Quantification of F4/80 + macrophages, CD11b + macrophages, and Ly6G + neutrophils per high power field (×200) (*n* = 6/group) (mean ± SD, *p < 0.05)

Because acute liver injury is intimately related to inflammatory response, KC relative cytokines and chemokines gene induction were detected in different groups by qRT-PCR. Livers from hyperglycemic mice treated with APAP showed significantly higher levels of proinflammatory TNF-α and IL-6, but lower levels of anti-inflammatory IL-10 gene induction compared to APAP treatment alone (Fig. 2a). These results were further confirmed by similar serum TNF-α, IL-6, and IL-10 levels (Fig. 2b). We furtherly evaluated the effect of hyperglycemia on regulating peripheral macrophage and neutrophil infiltration by immunohistochemical staining. Interestingly, hyperglycemia significantly increased the number of F4/80 + total intrahepatic macrophages, CD11b + infiltrating macrophages, and Ly6G + neutrophils in APAP-challenged livers (Fig. 2c–h).

### Hyperglycemia mediates KC M1/M2 polarization in response to APAP treatment

**Fig. 3:**
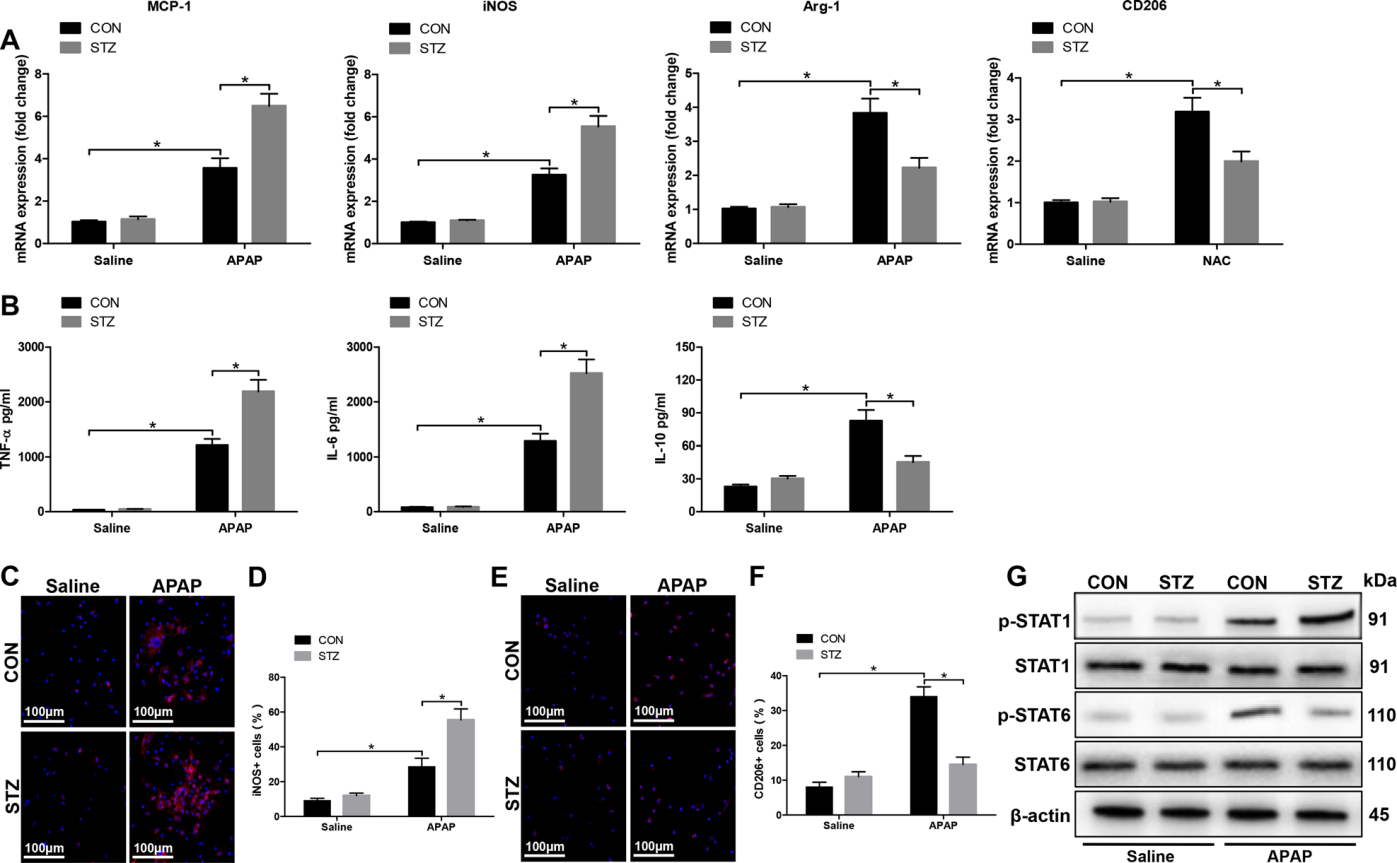
Hyperglycemia mediates KC M1/M2 polarization in response to APAP treatment. Hyperglycemic (streptozotocin, STZ) and control (CON) mice were prepared as described in “Materials and methods” section. APAP-induced acute liver injury or a sham procedure was performed. KCs were isolated from different experimental groups. **a** M1 markers (MCP-1 and iNOS) and M2 markers (Arg-1 and CD206) of gene induction were analyzed by quantitative RT-PCR (*n* = 3/group). **b** Isolated KCs from different experimental groups were cultured for 6 h, and TNF-α, IL-6, and IL-10 protein were measured in the culture supernatant by ELISA (*n* = 3/group). **c–f** Immunofluorescence staining of iNOS and CD206 in KCs (original magnification ×200). DAPI was used for nuclear staining. Representative of three experiments. Ratio of iNOS + and CD206 + cells in different experimental groups (*n* = 3/group). **g** Intracellular p-STAT1, p-STAT6, and β-actin protein levels were measured by western blot. Representative of three experiments (mean ± SD, *p < 0.05)

Macrophages can be broadly classified into M1 (proinflammatory) and M2 (anti-inflammatory) subtypes based on function^18^. Thus, we explored the role of hyperglycemia in affecting KC M1/M2 polarization. KCs isolated from hyperglycemic mice posttreated with APAP exhibited higher levels of MCP-1 and inducible NO synthase (iNOS) but much lower levels of Arg1 and CD206 gene induction compared to APAP treatment alone (Fig. 3a). We next analyzed the levels of TNF-α, IL-6, and IL-10 in KCs culture supernatant by ELISA. As shown in Fig. 3b, KCs isolated from hyperglycemic mice posttreated with APAP secreted higher levels of the proinflammatory cytokines TNF-α and IL-6, and lower levels of the anti-inflammatory cytokine IL-10. Hyperglycemia significantly increased the number of KCs positive for iNOS (M1 marker) post APAP treatment compared to APAP alone, but decreased the number of KCs positive for CD206 (M2 marker) (Fig. 3c–f). Furthermore, as shown by western blot in Fig. 3g, hyperglycemic KCs were marked by increased activation of STAT1 but decreased STAT6 activation post APAP treatment. These results showed that hyperglycemia augmented KC M1 polarization and inhibited KC M2 polarization post APAP treatment.

### Hyperglycemia mediates KC M1/M2 polarization by inducing ROS production against APAP-induced acute liver injury

**Fig. 4:**
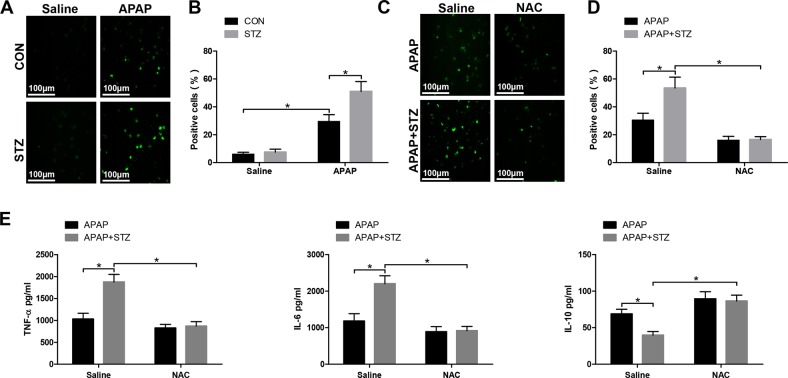
Hyperglycemia induces ROS production in KC in response to APAP-mediated acute liver injury. Mice were subjected to STZ pretreatment and APAP administration as described in “Materials and methods” section. KCs were isolated from different experimental groups. **a** ROS production was detected by CarboxyH2DFFDA in KCs (original magnification ×200). Representative of three experiments. Positive green fluorescent-labeled cells were counted blindly in 10 HPF/section (×200). **b** Quantification of ROS-producing KCs (green) per high power field (×200) (*n* = 3/group). Both CON and STZ mice were pretreated with ROS antagonist NAC or saline in vivo prior to APAP treatment. **c** KCs were isolated from different experimental groups. ROS production was detected by CarboxyH2DFFDA in KCs (original magnification ×200). Representative of three experiments. Positive green fluorescent-labeled cells were counted blindly in 10 HPF/section (×200). **d** Quantification of ROS-producing KCs (green) per high power field (×200) (*n* = 3/group). **e** Isolated KCs from different experimental groups were cultured for 6 h, and TNF-α, IL-6, and IL-10 protein were measured in the culture supernatant by ELISA (*n* = 3/group) (mean ± SD, *p < 0.05)

**Fig. 5:**
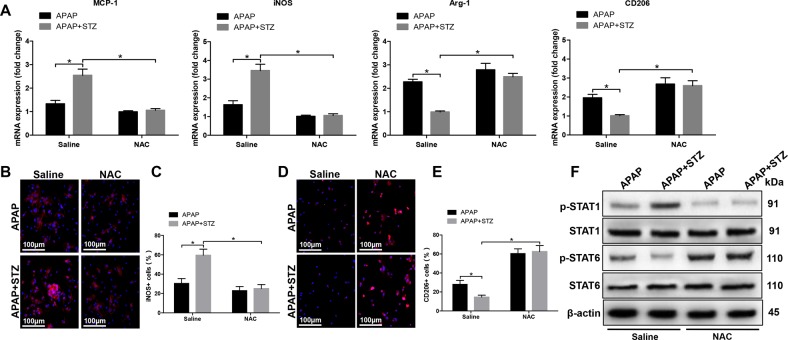
Hyperglycemia mediates KC M1/M2 polarization by inducing ROS production against APAP-induced acute liver injury. Mice were subjected to STZ pretreatment and APAP administration as described in “Materials and methods” section. Both CON and STZ mice were pretreated with ROS antagonist NAC or saline in vivo prior to APAP treatment. **a** KCs were isolated from different experimental groups. M1 markers (MCP-1 and iNOS) and M2 markers (Arg-1 and CD206) of gene induction were analyzed by quantitative RT-PCR (*n* = 3/group). **b–e** Immunofluorescence staining of iNOS and CD206 in KCs (original magnification ×200). DAPI was used for nuclear staining. Representative of three experiments. Ratio of iNOS + and CD206 + cells in different experimental groups (*n* = 3/group). **f** Intracellular p-STAT1, p-STAT6, and β-actin protein levels were measured by western blot. Representative of three experiments (mean ± SD, *p < 0.05)

Many evidences illustrated that hyperglycemia was related to ROS generation^14,19^. Based on these findings, we detected the levels of ROS in KCs from hyperglycemic mice post APAP treatment. The result showed that hyperglycemia significantly increased KCs ROS production (Fig. 4a, b). ROS inhibitor NAC was used to further study the functional role of ROS production in mediating hyperglycemic KC polarization. As shown in Fig. 4c–e, NAC significantly inhibited ROS production, decreased proinflammatory cytokines TNF-α and IL-6 and increased anti-inflammatory cytokines IL-10 secretion by KCs from hyperglycemic mice post APAP treatment compared to KCs from normoglycemic mice posttreated with APAP. Furthermore, KCs isolated from hyperglycemic mice posttreated with APAP and NAC exhibited lower levels of MCP-1 and iNOS but much higher levels of Arg1 and CD206 gene induction compared to APAP treatment alone (Fig. 5a). Similarly, NAC significantly decreased the number of KCs positive for iNOS (M1 marker) from hyperglycemic mice post APAP treatment compared to APAP alone, but increased the number of KCs positive for CD206 (M2 marker) (Fig. 5b–e). Furthermore, as shown by western blot in Fig. 5f, NAC decreased STAT1 activation but increased STAT6 activation in KCs from hyperglycemic mice treated with APAP. These results showed that ROS production promoted KC M1 polarization and inhibited KC M2 polarization in hyperglycemic mice post APAP treatment.

### Hyperglycemia mediates KC ROS production by inactivating AMPK and inducing PI3K/AKT signaling pathway activation in response to APAP treatment

**Fig. 6:**
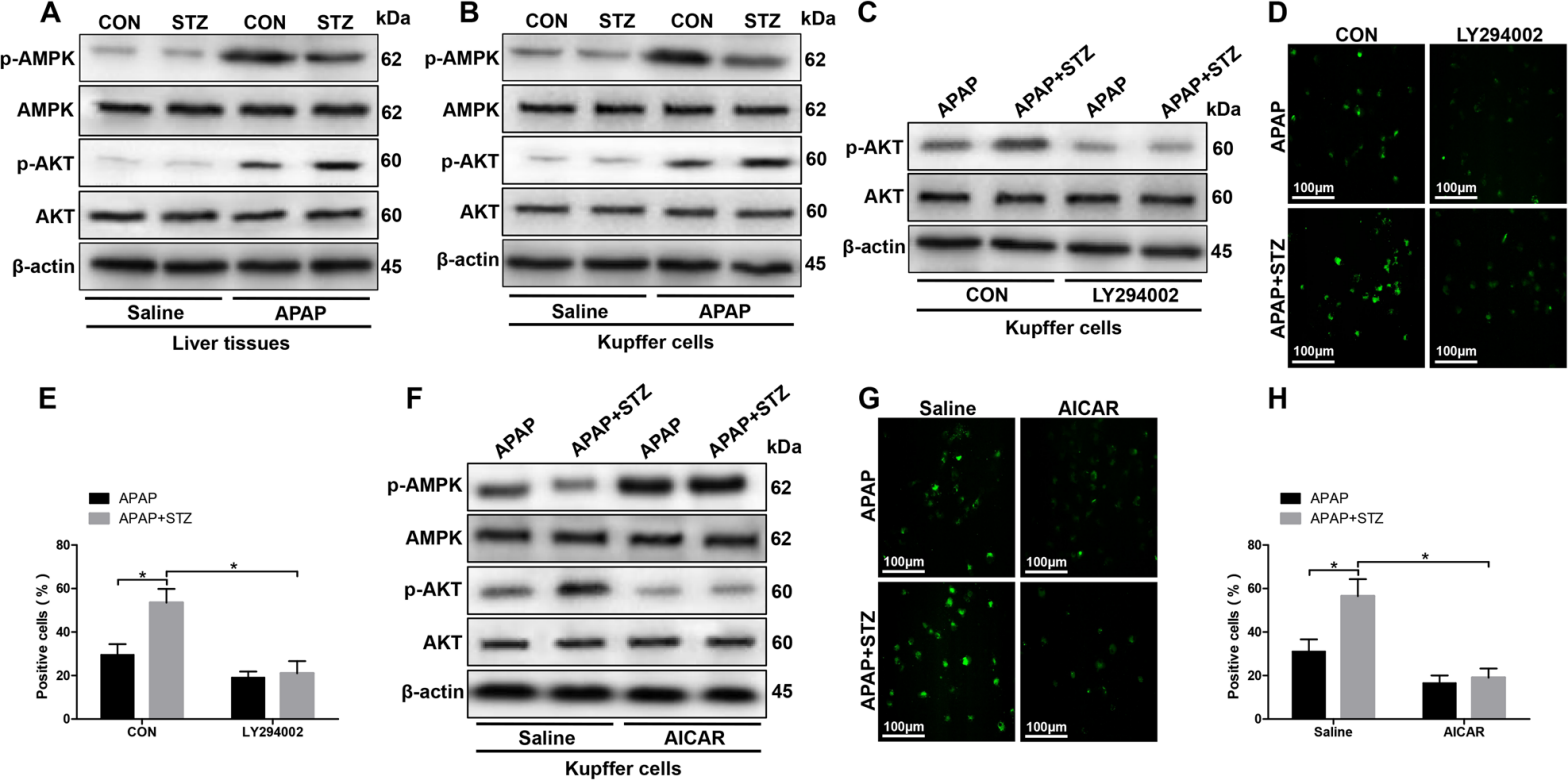
Hyperglycemia mediates KC ROS production by inactivating AMPK and activating PI3K/AKT signaling pathway. Mice were subjected to STZ pretreatment and APAP administration as described in “Materials and methods” section. KCs were isolated from different experimental groups. **a** p-AMPK, p-AKT, and β-actin protein levels in liver tissues from different groups were measured by western blot. Representative of three experiments. **b** p-AMPK, p-AKT, and β-actin protein levels in KCs isolated from different groups were measured by western blot. Representative of three experiments. **c** KCs isolated from APAP and STZ + APAP mice were treated with PI3K antagonist LY294002 (20 µM) for 6 h in vitro. Intracellular p-AKT, and β-actin protein levels were measured by western blot. Representative of three experiments. **d** ROS production was detected by CarboxyH2DFFDA in KCs (original magnification ×200). Positive green fluorescent-labeled cells were counted blindly in 10 HPF/section (×200). **e** Quantification of ROS-producing KCs (green) per high power field (×200) (*n* = 3/group). Both CON and STZ mice were pretreated with AMPK activator (AICAR) (AICAR, 100 mg/kg, i.p.) once a day for 7 days prior to TAA administration or saline in vivo prior to APAP treatment. **f** Intracellular p-AMPK, p-AKT, and β-actin protein levels were measured by western blot. Representative of three experiments. **g** ROS production was detected by CarboxyH2DFFDA in KCs (original magnification ×200). Positive green fluorescent-labeled cells were counted blindly in 10 HPF/section (×200). **h** Quantification of ROS-producing KCs (green) per high power field (×200) (*n* = 3 /group) (mean ± SD, *p < 0.05)

**Fig. 7:**
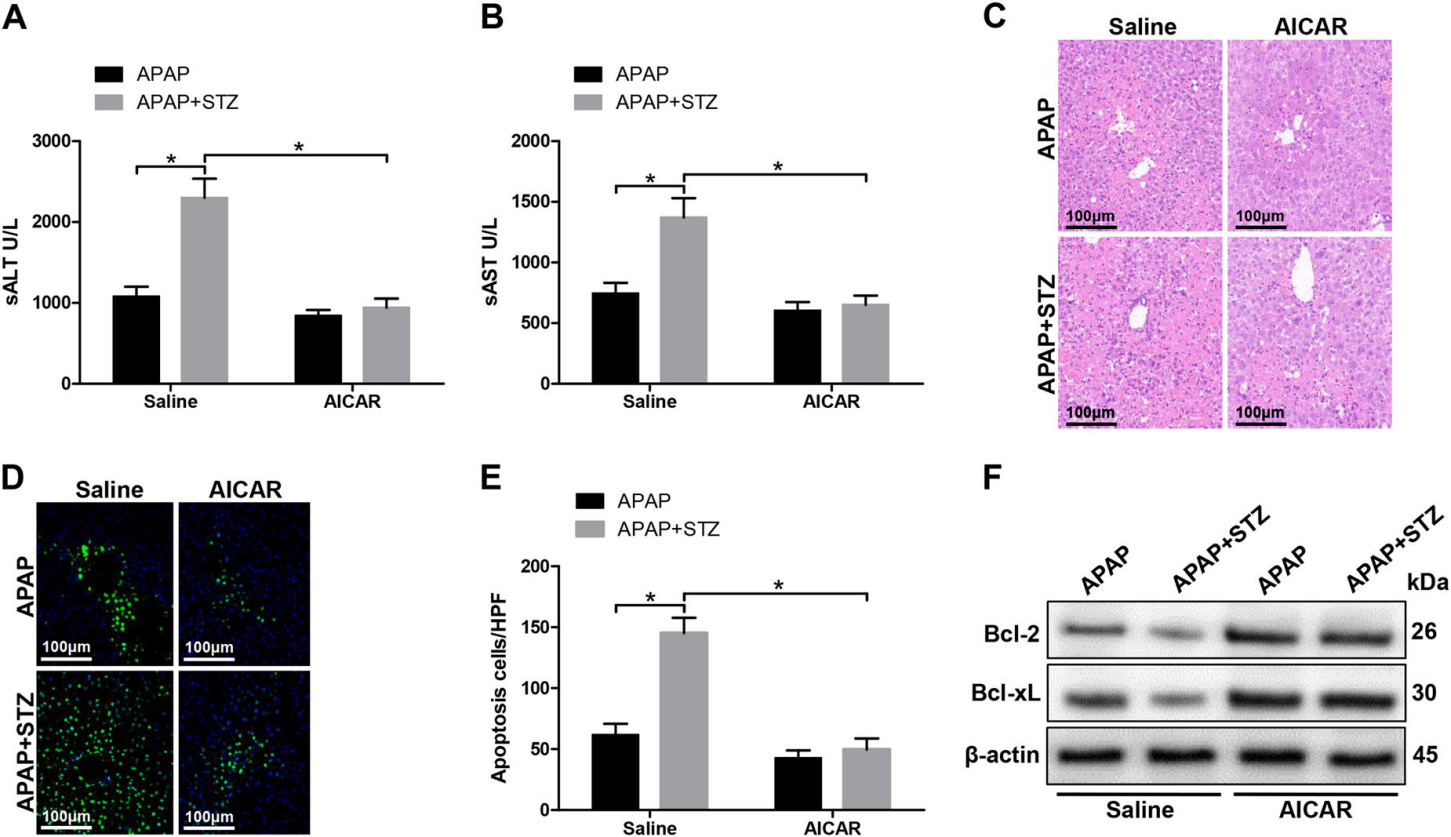
AMPK activator (AICAR) attenuates APAP-induced acute liver injury under hyperglycemia. Mice were subjected to STZ pretreatment and APAP administration as described in “Materials and methods” section. Both CON and STZ mice were pretreated with AMPK activator (AICAR) or saline in vivo prior to APAP treatment. **a**, **b** Liver injury was evaluated 24 h post APAP treatment in terms of serum AST and ALT levels (*n* = 6/group). **c** Liver injury was evaluated in terms of liver histopathology. Representative of six mice/group. **d** TUNEL staining of liver sections (original magnification ×200). DAPI was used for nuclear staining. Representative of six mice/group. **e** The ratio of TUNEL-positive cells in different experimental groups (*n* = 6/group). **f** Bcl-2, Bcl-xL, and β-actin protein levels were measured by western blot. Representative of three experiments (mean ± SD, *p < 0.05)

**Fig. 8:**
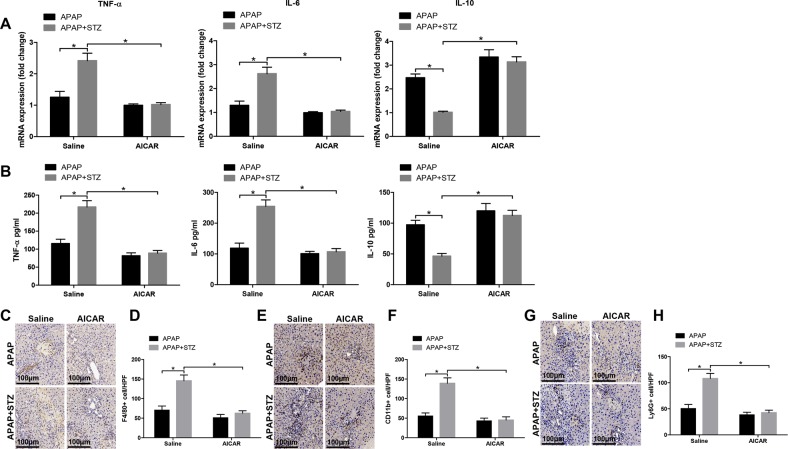
AMPK activator (AICAR) inhibites inflammatory response against APAP-exposed acute liver injury. Mice were subjected to STZ pretreatment and APAP administration as described in “Materials and methods” section. Both CON and STZ mice were pretreated with AMPK activator (AICAR) or saline in vivo prior to APAP treatment. **a** Inflammatory gene expression was measured by quantitative RT-PCR (*n* = 6/group). **b** Serum levels of inflammatory cytokines were measured by ELISA (*n* = 6/group). **c-h** F4/80 + macrophages, CD11b + macrophages, and Ly6G + neutrophils infltration in liver was detected by immunohistochemical staining (original magnification ×200). Representative of six mice/group. Quantification of F4/80 + macrophages, CD11b + macrophages, and Ly6G + neutrophils per high power field (×200) (*n* = 6/group) (mean ± SD, *p < 0.05)

To examine the mechanism of hyperglycemia mediating ROS production, we detected the protein levels of p-AMPK and p-AKT, which have been demonstrated associated with ROS generation, in liver and KCs from experimental groups^20,21^. As shown in Fig. 6a, significantly lower protein levels of p-AMPK and higher protein levels of p-AKT were found in liver tissues from hyperglycemic mice posttreated with APAP. Similarly, decreased protein levels of p-AMPK and increased protein levels of p-AKT were also found in KCs from hyperglycemic mice posttreated with APAP (Fig. 6b). To further determine the role of PI3K in regulating hyperglycemic KCs ROS production, we employed PI3K antagonist LY294002 in vitro. Indeed, LY294002 effectively inhibited AKT activation (Fig. 6c). Furthermore, LY294002 significantly inhibited hyperglycemic KCs ROS production posttreated with APAP (Fig. 6d, e). Afterward, AMPK activator AICAR was utilized to determine the relationship between the AMPK and PI3K/AKT signaling pathway. Indeed, AICAR significantly increased protein levels of p-AMPK and decreased protein levels of p-AKT in KCs isolated from hyperglycemic mice post APAP treatment (Fig. 6f). Moreover, AICAR notably inhibited hyperglycemic KCs ROS production posttreated with APAP (Fig. 6g, h). Interestingly, in vivo AMPK activation alleviated the traumatic role of hyperglycemia in APAP-induced liver injury, as evidenced by lower serum ALT and AST levels (Fig. 7a, b), decreased liver architecture damage (Fig. 7c), and decreased hepatocellular apoptosis (Fig. 7d–f) compared to APAP treatment alone. Finally, we detected KC relative cytokines gene induction in different groups by qRT-PCR. Hyperglycemic livers from APAP mice treated with AICAR showed significantly lower levels of proinflammatory TNF-α and IL-6, but higher levels of anti-inflammatory IL-10 gene induction compared to APAP treatment alone (Fig. 8a). These results were further confirmed by similar serum TNF-α, IL-6, and IL-10 levels (Fig. 8b). We furtherly evaluated the effect of AICAR on regulating peripheral macrophage and neutrophil infiltration by immunohistochemical staining. Interestingly, AICAR significantly decreased the number of F4/80 + total intrahepatic macrophages, CD11b + infiltrating macrophages, and Ly6G + neutrophils in APAP-challenged livers (Fig. 8c–h).

## **Discussion**

Diabetes mellitus/hyperglycemia is a major public health problem worldwide, which seriously impairs the quality of life of the patients^19,22^. Chronically untreated diabetes causes various macro- and micro-vascular complications, such as atherosclerosis^23^, diabetic nephropathy^24^, diabetic retinopathy^25^, and neural damage^19^. Our study addressed the question whether and how hyperglycemia impacted APAP-induced acute liver injury, focusing on its effects on the proinflammatory activation of the liver-resident macrophages (KCs). To the best of our knowledge, this is the first study to demonstrate that hyperglycemia exacerbates APAP-induced acute liver injury by promoting liver-resident macrophage proinflammatory response via AMPK/PI3K/AKT-mediated oxidative stress.

APAP is a commonly used nonprescription analgesic and antipyretic in many developed countries^4^. However, APAP overdose could cause liver injury in animals and humans by inducing mitochondria damage and subsequent necrosis in hepatocytes^26^. Excessive production of ROS was the main pathogenesis of APAP overdose, which was mediated by N-acetyl-p-benzoquinone imine, a metabolite of APAP^27^. Study demonstrated APAP could trigger an hepatic inflammatory response, including activation of resident macrophages and lymphocytes^28–30^. Other studies showed hyperglycemia aggravated hepatic ischemia and reperfusion injury due to hyper-inflammatory immune activation in KCs^2,3^. These studies showed KCs play a vital role in acute liver injury. However, the effect of hyperglycemia on KCs against APAP-induced liver inflammation remains unknown.

KCs exhibit a tremendous plasticity, depending on the local metabolic and immune environment. They can express a range of polarized phenotypes, from the proinflammatory M1 phenotype to the anti-inflammatory M2 phenotype^7^. M1 activated macrophages are characterized by increased expression of transcription factors, such as STAT1 and interferon-regulatory factor 5 (IRF-5)^31^, and proinflammatory cytokines, including TNF-α, IL-6, IL-12, and (iNOS)^32^. While M2 activated macrophages exhibit high expression of transcription factors, including STAT6, IRF-4, and PPARγ^31^, and anti-inflammatory mediators, such as IL-10 and IL-1 decoy receptor^32^. Follow-up studies indicated that inactivation of these tissue macrophages with gadolinium chloride and dextran sulfate attenuated the moderate liver injury after APAP treatment^33^. A similar protection together with elimination of nitrotyrosine staining was reported after treatment with gadolinium chloride in a mouse model of APAP-induced liver damage^34^. Because gadolinium chloride and related compounds functionally inactivated KCs, that is, mainly impaired their capacity to generate rROS^35,36^. These data indicated that KCs could be the dominant source of ROS formation after APAP overdose^34,37^.

ROS play a pivotal role in the development, growth, and differentiation of multicellular organisms. They are kept in cells at a baseline level that supports cellular proliferation and metabolism, but are also used as key signal transduction molecules that regulate many important metabolic and regulatory pathways in cells^38^. Recent studies showed that oxidative stress was a major mediator that underlied diabetic complications^12,39,40^. Studies showed hyperglycemia triggered ROS formation in macrophages^13,19^. Monocytes were attracted by MCP-1 secreted by endothelium under diabetes^13^. Transmigrated monocytes differentiated into macrophages and produced increased ROS levels, aggravating inflammation and tissues injury^13^. Other studies also showed that hyperglycemia increased ROS production by causing mitochondrial dysfunction in macrophages and aberrant activation of cytoplasmic NADPH oxidases (NOX)^16,17^. In contrast to well-regulated ROS production in anti-microbial response, metabolically generated ROS in diabetic macrophages is more erratic and dysregulated. Its production was associated with promotion of M1-like macrophage phenotype that favors the progression of diabetic complications^41^. It was hypothesized that the metabolism of APAP triggered the oxidant stress^42^. Other studies had confirmed that ROS production induced by mitochondrial oxidative stress played an important role in APAP-induced hepatoxic damage^43,44^.

According to the above studies, we tested how hyperglycemia impacted APAP-induced acute liver injury, focusing on its effects on the proinflammatory activation of KCs. We first detected the levels of ROS in KCs from hyperglycemic mice post APAP treatment. The result showed hyperglycemia increased ROS production and inflammatory response of KCs against APAP-induced acute liver injury. ROS inhibition by its antagonist NAC effectively suppressed ROS production and inflammatory response of KCs from hyperglycemic mice post APAP treatment. Furthermore, we hypothesized that AMPK could be associated with the PI3K/AKT signaling pathway in KCs from hyperglycemic mice post APAP treatment according to the KEGG database. Therefore, we measured protein levels of p-AMPK, AMPK, p-AKT, and AKT. We found hyperglycemia increased p-AMPK protein expression while decreasing p-AKT protein expression. PI3K inhibition by its antagonist LY294002 or AMPK activation by its agonist AICAR inhibited AKT activation, and subsequently, inhibited ROS production in KCs, leading to ultimately reduced APAP-induced liver injury in the hyperglycemic mice. These results showed hyperglycemia increased ROS production by inhibiting AMPK activation and inducing PI3K/AKT signaling pathway activation.

In conclusion, our results demonstrated that hyperglycemia exacerbated APAP-induced acute liver injury by promoting liver-resident macrophage proinflammatory response via AMPK/PI3K/AKT-mediated oxidative stress. These findings indicated that inhibition of oxidative stress could be a novel therapeutic approach to mitigate APAP-induced hepatotoxicity and liver injury in hyperglycemia.

## Materials and methods

### Animals

Male wild-type C57BL/6 J mice (6–8 weeks old) were purchased from the Laboratory of Animal Resources of Nanjing Medical University and were housed under specific pathogen-free conditions with access to properly sterilized water and food. This study was performed in strict accordance with the recommendations in the protocol (number NMU08–092) approved by the Institutional Animal Care and Use Committee of Nanjing Medical University.

### Mouse hyperglycemia and APAP-induced acute liver injury model

Streptozotocin (STZ) or vehicle control (sodium citrate buffer) was injected intraperitoneally (i.p.) into separate groups of 6-week-old mice at 40 mg/kg for five consecutive days. Blood glucose levels from the tail vein were tested at day 14 (9 days after the last shot). Mice with blood glucose over 300 mg/dL were considered hyperglycemic (STZ group). The vehicle control group (CON group) was subject to the same intraperitoneal injection procedure, but with sodium citrate buffer. The mice were intraperitoneally injected with a dose of 400 mg/kg APAP (Sigma, St. Louis, MO, USA) dissolved in saline. The control mice received the same volume of saline via intraperitoneal injection. The mice were killed 24 h after APAP treatment. Serum and liver samples were collected. Tissues were stored continuously in liquid N_2_. Sections from the dissected livers were also fixed in 10% neutral buffered formalin for histological analysis.

### Hepatocellular function assay

Blood samples were collected and centrifuged to obtain serum for analysis. Serum levels of ALT and AST were detected by an automated chemical analyzer (Olympus Company, Tokyo, Japan).

### TUNEL staining

TUNEL staining of liver tissues was performed using a fluorescent detection kit (Roche, Switzerland) according to the manufacturer’s instructions. Briefly, paraffin sections of hepatic tissues (4 µm thickness) were deparaffinated in toluene and then dehydrated by a graded series of ethanol solutions (ethanol 100, 95, 90, 80, and 70%) and washed in distilled water. They were then incubated with 20 µg/ml proteinase K solution (Roche, Switzerland) for 25 min at 37 °C. The tissues were then washed with PBS three times for 3 min. After washing with PBS, the tissues were then incubated with permeabilize working solution at 37 °C for 20 min. The tissues were then washed with PBS three times for 3 min. Next, the tissues were incubated with TUNEL solution (TdT: dUTP = 1: 9) at 37 °C for 2 h in a moist and dark environment. The tissues were then washed with PBS three times for 3 min and incubated with DAPI solution at 37 °C for 10 min in a dark environment. Then the tissues were washed with PBS three times for 3 min. Sections were photographed by Fluorescent Microscopy. DAPI glows blue by UV excitation wavelength 330–380 nm and emission wavelength 420 nm; FITC glows green by excitation wavelength 465–495 nm and emission wavelength 515–555 nm. The nucleus is blue by labeling with DAPI. Tunel assay kit is from Roch and labeled with FITC. Positive apoptosis cells are green.

### Histopathology, immunohistochemical staining, and immunofluorescence staining

Liver tissues were collected and stained with hematoxylin and eosin, and light microscopy was used to observe inflammation and tissue damage. Liver macrophages and neutrophils were detected using primary rat anti-mouse F4/80, CD11b, and Ly6G mAb, respectively (BD Biosciences, San Jose, CA, USA). The secondary, biotinylated goat anti-rat IgG (Vector, Burlingame, CA, USA) was incubated with immunoperoxidase (ABC Kit, Vector), according to the manufacturer’s instruction. Positive cells were counted blindly in 10 HPF/section. iNOS and CD206 in KCs were identified by immunofluorescence using rabbit anti-mouse iNOS mAb and anti-mouse CD206 mAb (Cell Signaling Technology, MA, USA). After incubation with secondary goat anti-mouse Texas Red-conjugated IgG (Sigma, St.Louis, MO, USA), KCs were premounted with VECTASHIELD medium with DAPI (Vector). Negative control slided with the primary antibodies omitted were included in all assays. Positive cells were blindly observed in 10 HPF/section (×200).

### Cell isolation and culture

KCs were isolated as previously described^45^. Briefly, livers were perfused in situ via the portal vein with calcium- and magnesium-free HBSS supplemented with 2% heat-inactivated FBS, followed by 0.27% collagenase IV (Sigma, St. Louis, MO). Perfused livers were dissected and teased through 70 mm nylon mesh cell strainers (BD Biosciences, San Diego, CA), followed by suspension in 40 ml of DMEM supplemented with 10% FBS, 10 mM HEPES, 2 mM GlutaMax, 100 U/ml penicillin, and 100 mg/ml streptomycin for 15 min at 37 °C. Nonadherent cells were removed by replacing the culture medium. The adherent cells were used for further ex vivo experiments. KCs were cultured in vitro for 6 h, and then cells or supernatants were collected for further analysis. For M2 polarization, KCs isolated from mice were treated with 10 ng/ml IL-4 (Sigma, Saint Louis, MO, USA).

### Western blot analysis

Liver tissues and cellular proteins were extracted with ice-cold lysis buffer [1% Triton X-100, 0.5% sodium deoxycholate, 0.1% SDS, 10% glycerol, 137 mM sodium chloride, and 20 mM Tris (pH 7.4)]. Proteins (20 µg) were separated by 10% SDS-PAGE and transferred to polyvinylidene difluoride nitrocellulose membranes. Phospho-STAT1, STAT1, phospho-STAT6, STAT6, phospho-AMPK, AMPK, phospho-AKT, AKT, Bcl-2, Bcl-xl, and β-actin rabbit mAbs (Cell Signaling Technology, MA, USA) were used. HRP-conjugated goat anti-rabbit IgG (Cell Signaling Technology, MA, USA) was used as the secondary antibody.

### Quantitative RT-PCR analysis

Total RNA was extracted from frozen liver tissues and cells using TRIZOL reagent (Invitrogen, Carlsbad, CA, USA) and was reverse-transcribed into cDNA using the Transcriptor First Strand cDNA Synthesis Kit (Roche, Indianapolis, IN, USA). Quantitative real-time PCR was performed using SYBR green (Roche, Indianapolis, IN, USA) on a StepOnePlus Real-Time PCR System (Applied Biosystems, Foster City, CA). PCR cycling conditions were as follows: 95 °C for 30 s; 40 cycles of 95 °C for 5 s and 60 °C for 30 s; and dissociation at 95 °C for 15 s, 60 °C for 60 s, and 95 °C for 15 s. qRT-PCR reactions were all repeated three times. The expression levels of target genes and the results were normalized against HPRT expression. Primer sequences used for the amplification were shown in Table 1.

**Table 1:** Primer sequences for the amplification

Gene	Forward primer (5′ → 3′)	Reverse primer (5′ → 3′)
TNF-α	5′-GACGTGGAACTGGCAGAAGAG-3′	5′-TTGGTGGTTTGTGAGTGTGAG-3′
IL-6	5′-CCAAGAGGTGAGTGCTTCCC-3′	5′-CTGTTGTTCAGACTCTCTCCCT-3′
IL-10	5′-GCTCTTACTGACTGGCATGAG-3′	5′-CGCAGCTCTAGGAGCATGTG-3′
MCP-1	5′-TTAAAAACCTGGATCGGAACCAA-3′	5′-GCATTAGCTTCAGATTTACGGGT-3′
iNOS	5′-GTTCTCAGCCCAACAATACAAGA-3′	5′-GTGGACGGGTCGATGTCAC-3′
Arg-1	5′-CTCCAAGCCAAAGTCCTTAGAG-3′	5′-AGGAGCTGTCATTAGGGACATC-3′
CD206	5′-CTCTGTTCAGCTATTGGACGC-3′	5′-CGGAATTTCTGGGATTCAGCTTC-3′
HPRT	5′-TCAGTCAACGGGGGACATAAA-3′	5′-GGGGCTGTACTGCTTAACCAG-3′

### ROS assay

ROS production in the KCs was measured using the Carboxy-H2DFFDA kit (Thermo Fisher Scientific, Waltham, MA). ROS production by the KCs was analyzed and quantified by confocal microscopy (ZEISS, Oberkochen, Germany) according to the manufacturer’s instructions.

### ELISA

TNF-α, IL-6, and IL-10 levels in serum or cell culture supernatants were measured using an ELISA kit (eBiosciences, San Diego, CA, USA) according to the manufacturer’s protocols.

### Statistical analysis

Data were expressed as the mean ± SD and analyzed using the Permutation *t*-test and Pearson correlation. Multiple group comparisons were performed using one-way ANOVA followed by Bonferroni’s post hoc test. All analysis were performed using Stata software (version 11.0). *P* *<* 0.05 (two-tailed) was considered statistically significant.
